# Insight into Potential Probiotic Markers Predicted in *Lactobacillus pentosus* MP-10 Genome Sequence

**DOI:** 10.3389/fmicb.2017.00891

**Published:** 2017-05-22

**Authors:** Hikmate Abriouel, Beatriz Pérez Montoro, Carlos S. Casimiro-Soriguer, Antonio J. Pérez Pulido, Charles W. Knapp, Natacha Caballero Gómez, Sonia Castillo-Gutiérrez, María D. Estudillo-Martínez, Antonio Gálvez, Nabil Benomar

**Affiliations:** ^1^Área de Microbiología, Departamento de Ciencias de la Salud, Facultad de Ciencias Experimentales, Universidad de JaénJaén, Spain; ^2^Centro Andaluz de Biología del Desarrollo – Consejo Superior de Investigaciones Cientificas, Universidad Pablo de OlavideSevilla, Spain; ^3^Department of Civil and Environmental Engineering, University of StrathclydeGlasgow, United Kingdom; ^4^Área de Estadística e Investigación Operativa, Departamento de Estadística e Investigación Operativa, Facultad de Ciencias Experimentales, Universidad de JaénJaén, Spain

**Keywords:** Aloreña table olives, *Lactobacillus pentosus*, probiotics, *in silico* analysis, carbohydrate metabolism, host interaction

## Abstract

*Lactobacillus pentosus* MP-10 is a potential probiotic lactic acid bacterium originally isolated from naturally fermented Aloreña green table olives. The entire genome sequence was annotated to *in silico* analyze the molecular mechanisms involved in the adaptation of *L. pentosus* MP-10 to the human gastrointestinal tract (GIT), such as carbohydrate metabolism (related with prebiotic utilization) and the proteins involved in bacteria–host interactions. We predicted an arsenal of genes coding for carbohydrate-modifying enzymes to modify oligo- and polysaccharides, such as glycoside hydrolases, glycoside transferases, and isomerases, and other enzymes involved in complex carbohydrate metabolism especially starch, raffinose, and levan. These enzymes represent key indicators of the bacteria’s adaptation to the GIT environment, since they involve the metabolism and assimilation of complex carbohydrates not digested by human enzymes. We also detected key probiotic ligands (surface proteins, excreted or secreted proteins) involved in the adhesion to host cells such as adhesion to mucus, epithelial cells or extracellular matrix, and plasma components; also, moonlighting proteins or multifunctional proteins were found that could be involved in adhesion to epithelial cells and/or extracellular matrix proteins and also affect host immunomodulation. *In silico* analysis of the genome sequence of *L. pentosus* MP-10 is an important initial step to screen for genes encoding for proteins that may provide probiotic features, and thus provides one new routes for screening and studying this potentially probiotic bacterium.

## Introduction

The *Lactobacillus* genus belongs to the LAB group, which currently comprises of 222 species described in List of Prokaryotic Names with Standing in Nomenclature “LPSN”^1^ (February 2017). In this context, *Lactobacillus* represents a highly heterogeneous taxonomic group encompassing species with various physiological, biochemical and genetic characteristics that reflect their capacity to colonize many ecological niches and to respond to several environmental stresses (De Angelis and Gobbetti, 2004; Pot et al., 2014). Lactobacilli have been isolated from different sources [e.g., plants, foods, and the mucosal surfaces (i.e., from oral, gastrointestinal, and reproductive tracts) of mammalian hosts], and they have widely been used as starter cultures in food fermentations, due to their safe-history of use, and also as protective cultures because of their production of antimicrobial substances (e.g., bacteriocins, peroxide, diacetyl, among others) (Leroy and de Vuyst, 1999; Heller, 2001; Hansen, 2002; Holzapfel, 2002; Giraffa et al., 2010; Franz et al., 2011; Garrigues et al., 2013). Thus, the Food and Drug Administration and European Food Safety Authority certify some *Lactobacillus* species as Generally Recognized As Safe (GRAS) or having a Qualified Presumption of Safety (QPS), respectively (Bernardeau et al., 2008). Furthermore, many *Lactobacillus* species represent main components of the global probiotic market: *L. acidophilus*, *L. bulgaricus*, *L. plantarum*, *L. brevis*, *L. reuteri*, *L. johnsonii*, *L. casei*, *L. rhamnosus*, and *L. salivarius*. Specifically, some *L. pentosus* strains have exerted probiotic effects such as the acceleration of IgA secretion in saliva and the enhancement of IgA production in the small intestine (Kotani et al., 2010; Izumo et al., 2011), which have aroused great interest due to vegetal origin (Pérez Montoro et al., 2016). Generic mechanisms for underlying probiotic effects can be linked to taxonomic groups (genus or species); however, specific mechanisms tend to be strain-specific (Hill et al., 2014). As such, whole genome sequencing (WGS) remains the best way to better understand the genetic and metabolic potential of each species/strain, to demonstrate the plasticity of their phylogenetic relationships, metabolic pathways, adaptation, fitness and safety (Jolley and Maiden, 2010; Maiden et al., 2013).

*Lactobacillus pentosus* MP-10 is a potential probiotic LAB isolated from naturally fermented Aloreña green table olives (Abriouel et al., 2011) and has exhibited several probiotic capacities when tested *in vitro* such as good growth and survival capacities under simulated gastro-intestinal conditions, ability to auto-aggregate, and co-aggregate with pathogenic bacteria, adherence to intestinal and vaginal cell lines, antagonistic activity against pathogens and fermentation of several prebiotics and lactose (Pérez Montoro et al., 2016). However, the putative health-promoting capacities of this strain may depend on genetic characteristics and the interactions within its ecological niche (O’Sullivan et al., 2009); for this reason, the whole-genome sequence obtained by Abriouel et al. (2016) and the subsequent annotation will improve our knowledge about the functionality of this strain, its adaptation to the human gastrointestinal tract (GIT) and its interaction within the host. As such, we carried out *in silico* analysis of *L. pentosus* MP-10’s carbohydrate metabolism and the factors that affect their interaction with the host with the aim to identify genes as potential probiotic markers.

## Results and Discussion

### General Metabolic Features of a Probiotic *Lactobacillus pentosus* MP-10

**FIGURE 1:**
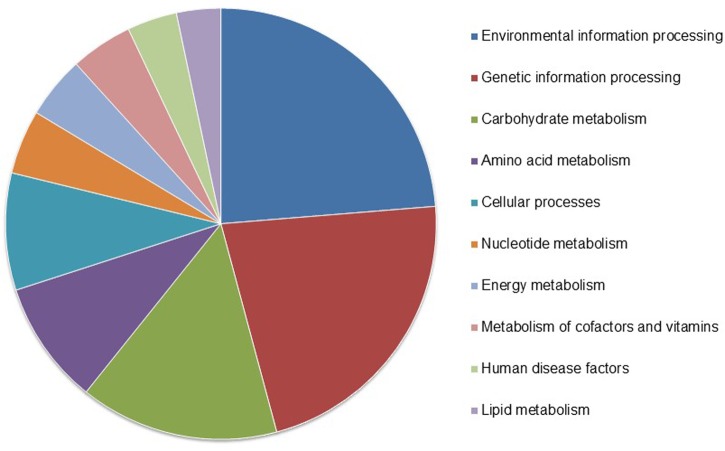
**BlastKOALA results of functional categories predicted in *Lactobacillus pentosus* MP-10 genome and their frequencies**.

**Figure 1** shows the frequency of KEGG functional annotations obtained by BlastKOALA (KEGG tool; last updated March 4, 2016), which assigned approximately half (45.7%) of the genes to KEGG annotations corresponding to environmental information processing (443 genes), genetic information processing (413 genes), carbohydrate metabolism (279), amino acid metabolism (173), cellular processes (164 genes), nucleotide metabolism (90 genes), energy metabolism (87 genes), metabolism of cofactors and vitamins (87 genes), human disease factors (70 genes), lipid metabolism (62 genes), among others.

To highlight the molecular mechanisms involved in the adaptation of *L. pentosus* MP-10 to the human GIT, we focused the *in silico* analysis on carbohydrate metabolism related to prebiotic utilization and the proteins involved in host interactions, since the adaptation of probiotics is mainly represented by the enrichment of mucus-binding proteins and enzymes involved in breakdown of complex carbohydrates (Ventura et al., 2012).

*In silico* analysis has some limitations related with the prediction accuracy which in turn depends on the algorithm used and the phenotype data from experiments (Ng and Henikoff, 2006); however, to avoid incorrect predictions all the annotations made in the present study were curated manually.

### Carbohydrate Metabolism Related with Prebiotic Utilization

Over 8% of the identified genes in *L. pentosus* MP-10 genome are involved in carbohydrate metabolism (279 of 3558 genes), which is similar to the most-studied bifidobacterial genomes and 30% higher than other gastrointestinal (GIT)-resident bacteria (Ventura et al., 2009). The abundance of carbohydrate metabolism genes in *L. pentosus* MP-10 is important with respect to its possible adaptation to the microhabitats of gastrointestinal environment and its interaction with human host, and thus may enhance its survival, competitiveness and persistence.

*Lactobacillus pentosus* MP-10 is a facultatively hetero-fermentative LAB, and its genome possesses genes for both the phosphoketolase and Embden-Meyerhof pathways (EMP). Thus, it can potentially ferment carbohydrates mainly via the EMP, utilizing glucose, and converting it to pyruvate and then to lactate (glycolysis). However, in the absence of six-carbon sugars (e.g., glucose, et al.), *L. pentosus* MP-10 would possibly ferment five-carbon carbohydrates such as xylose, xylulose, arabinose, or ribose via the phosphoketolase pathway (PK), as reported for other *L. pentosus* strains (Bustos et al., 2005). Analysis by BlastKOALA indicated that EMP (complete pathway), pentose phosphate pathway (PP) (both oxidative and non-oxidative complete pathways), and galactose degradation pathway (complete Leloir pathway) form the central core of carbohydrate metabolism in *L. pentosus* MP-10; however, the Entner-Doudoroff pathway (ED) appears incomplete.

**FIGURE 2:**
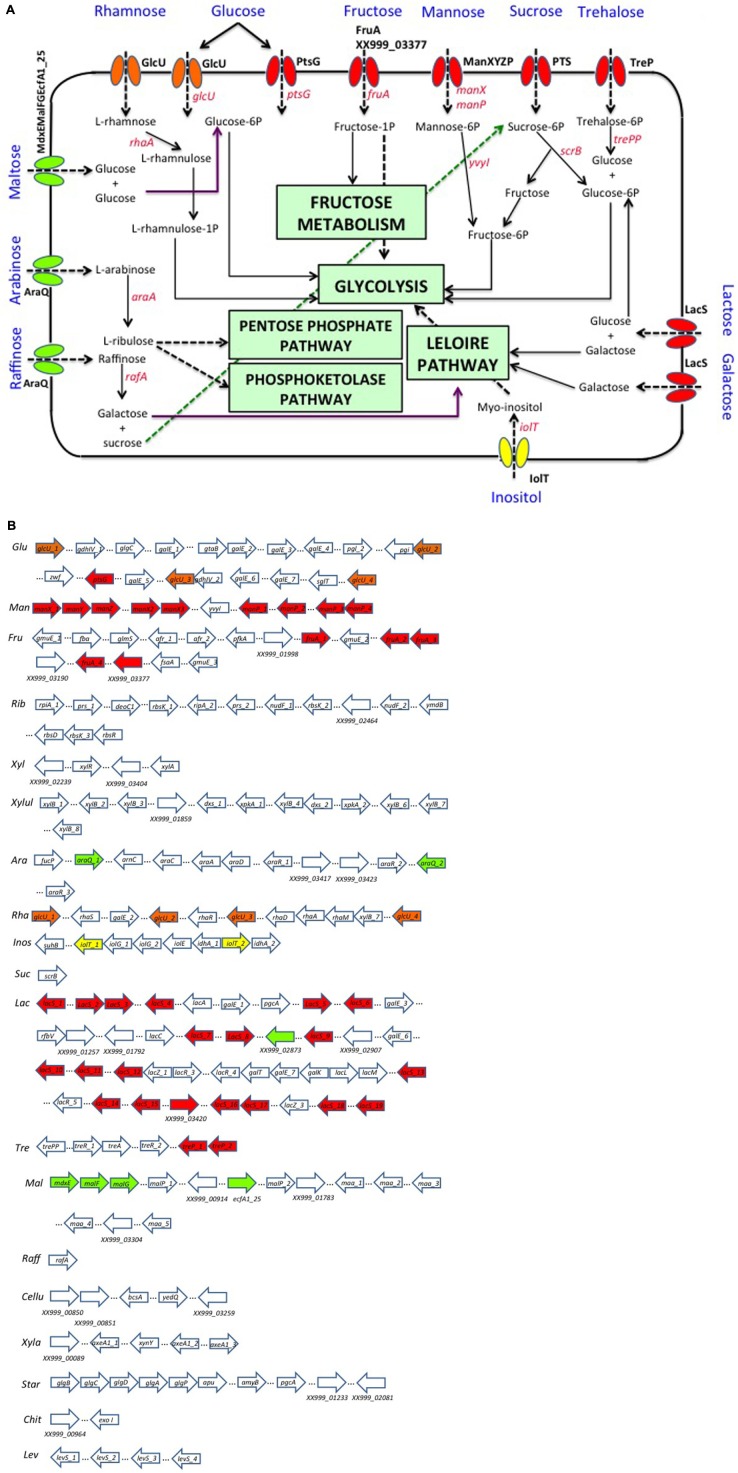
**Organization of gene clusters encoding proteins predicted to be involved in carbohydrate utilization as prebiotics by *L. pentosus* MP-10.** **(A)** Pathway reconstruction as predicted by genome annotation: PTS (phosphotransferase system), red; MFS (Major Facilitator Superfamily), yellow; ABC Transporter, green; GRP (Glucose/Ribose Porter Family), orange. **(B)** Genetic loci of interest: *Ara*, arabinose; *Cellu*, cellulose; *Chit*, chitin; *Fru*, fructose; *Glu*, glucose; *Inos*, inositol; *Lac*, lactose–galactose loci; *Lev*, levan; *Mal*, maltose; *Man*, mannose; *Raff*, raffinose; *Rha*, rhamnose; *Rib*, ribose; *Star*, starch; *Suc*, sucrose; *Tre*, trehalose; *Xyl*, xylose; *Xyla*, xylan; *Xylul*, xylulose.

*Lactobacillus pentosus* MP-10 has been shown to be able to ferment *in vitro* a variety of carbohydrates such as glucose, galactose, fructose, lactose, saccharose, and lactulose (Pérez Montoro et al., 2016). *In silico* analysis of the annotated genome sequence of *L. pentosus* MP-10 also predicted its capacity to ferment several simple carbohydrates of both five-carbon and six-carbon sugars such as mannose, inositol, ribose, arabinose, rhamnose, maltose, xylose, xylulose, and trehalose; furthermore, we also predicted its ability to use complex carbohydrates such as cellulose, xylan (hemicellulose), starch, raffinose, chitin, and levan (**Figure 2**). These carbohydrates can either be dietary compounds or carbon sources derived from the metabolism of the gastrointestinal microbiota (Korakli et al., 2002). Ultimately, 15 carbohydrate utilization pathways were predicted in *L. pentosus* MP-10 genome sequence: glycolysis/gluconeogenesis, citrate cycle, PP pathway, pentose, and glucuronate interconversions, fructose and mannose metabolism, galactose metabolism, ascorbate, and aldarate metabolism, starch and sucrose metabolism, amino sugar and nucleotide sugar metabolism, pyruvate metabolism, glyoxylate and dicarboxylate metabolism, propanoate metabolism, butanoate metabolism, C5-branched dibasic acid metabolism and inositol phosphate metabolism. As such, the wide repertoire of enzymes involved in the fermentation of various carbohydrate substrates is reflected in its relatively large genome size, which is also corroborated by the significantly abundant number of genes for the phosphoenolpyruvate- (PEP) dependent sugar phosphotransferase system (PTS) (77 genes) and the presence of specific genes or gene clusters involved in carbohydrate utilization by *L. pentosus* MP-10.

The possible adaptation and enrichment of *L. pentosus* MP-10 in GIT could be predicted by the presence of genes encoding various carbohydrate-modifying enzymes able to modify oligo- and polysaccharides. These enzymes are produced by intestinal microbial communities and are required for the metabolism of plant- and host-derived carbohydrates (e.g., cellulose, xylan, and pectin), since mammals have limited evolved abilities to hydrolyze complex polysaccharides for digestion (Cantarel et al., 2012). Among these enzymes, many were predicted in *L. pentosus* MP-10 genome and belong to several CAZY “Carbohydrate-Active Enzymes” families (**Table 1**): glycoside hydrolases or glycosylases (15 genes); hexosyl- (15 genes), pentosyl- (13 genes) and phospho-transferases (13 genes); and also isomerases (24 genes).

**Table 1:** Putative carbohydrate-modifying enzymes identified in the genome sequence of *Lactobacillus pentosus* MP-10.

	Enzyme	Gene	Gene ID	EC number	CAZy Family^∗^
**Hexosyltransferases**	Glycogen phosphorylase	*glgP*	XX999_00118	EC:2.4.1.1	GT35
	Maltose phosphorylase	*mapA*	XX999_00299	EC:2.4.1.8	GH65
	Cellulose synthase (UDP-forming)	*bcsA*	XX999_01782	EC:2.4.1.12	GT6
	1,4-alpha-glucan branching enzyme^∗∗^	*glgB*	XX999_01507	EC:2.4.1.18	GH13, GH57
	Starch synthase^∗∗^	*glgA*	XX999_00114	EC:2.4.1.21	GT5
	Poly(glycerol-phosphate) alpha-glucosyltransferase	*tagE*	XX999_00117	EC:2.4.1.52	GT4
	Alpha, alpha-trehalose phosphorylase	E2.4.1.64	XX999_01349	EC:2.4.1.64	GH65
	Peptidoglycan glycosyltransferase	*pbp2A*	XX999_01350	EC:2.4.1.129	GT51
	*N*-acetylglucosaminyldiphosphoundecaprenol	*tagA*	XX999_02448	EC:2.4.1.187	–
	*N*-acetyl-beta-D-mannosaminyltransferase		XX999_02762		
			XX999_02763		
		*murG*		EC:2.4.1.227	GT28
				EC:2.4.1.337	–
	Undecaprenyldiphospho-muramoylpentapeptide beta-*N*-acetylglucosaminyltransferase	*bgsB*	XX999_03361	EC:2.4.1.–	GH1, GH3, GH5, GH13, GH16, GH17, GH20, GH27, GH31, GH32, GH33, GH35, GH39, GH65, GH70, GH72, GH94, GH112, GH130
		*rfaB*	XX999_01483	EC:2.4.1.– 3.4.–.–	
	1,2-diacylglycerol 3-alpha-glucosyltransferase	*mrcA*	XX999_00670	EC:2.4.1.–	
	UDP-D-galactose:(glucosyl)LPS alpha-1,6-D-galactosyltransferase^∗∗^	*icaA*	XX999_02161	EC:2.4.1.–	GH1, GH3, GH5, GH13, GH16, GH17, GH20, GH27, GH31, GH32, GH33, GH35, GH39, GH65, GH70, GH72, GH94, GH112, GH130
		*cpoA*	XX999_01307		
			XX999_01219		
					GH1, GH3, GH5, GH13, GH16, GH17, GH20, GH27, GH31, GH32, GH33, GH35, GH39, GH65, GH70, GH72, GH94, GH112, GH130
			XX999_01806		
	Penicillin-binding protein 1A^∗∗^				
					GH1, GH3, GH5, GH13, GH16, GH17, GH20, GH27, GH31, GH32, GH33, GH35, GH39, GH65, GH70, GH72, GH94, GH112, GH130
	Poly-beta-1,6-*N*-acetyl-D-glucosamine synthase^∗∗^		XX999_01594		
	1,2-diacylglycerol-3-alpha-glucose alpha-1,2-galactosyltransferase^∗∗^		XX999_01308		
**Pentosyltransferases**	Adenine phosphoribosyltransferase	*apt*	XX999_01330	EC:2.4.2.7	GH10
	Hypoxanthine phosphoribosyltransferase	*hpt*	XX999_02067	EC:2.4.2.8	GH10
	Uracil phosphoribosyltransferase	*upp*	XX999_00627	EC:2.4.2.9	GH10
	Pyrimidine operon attenuation protein/uracil phosphoribosyltransferase	*pyrR*	XX999_02348	EC:2.4.2.9	GH10
	Orotate phosphoribosyltransferase	*pyrE*	XX999_01829	EC:2.4.2.10	GH10
	Amidophosphoribosyltransferase	*purF*	XX999_02638	EC:2.4.2.14	GH10
	ATP phosphoribosyltransferase	*hisG*	XX999_02631	EC:2.4.2.17	GH10
	Anthranilate phosphoribosyltransferase	*trpD*	XX999_02648	EC:2.4.2.18	GH10
	Xanthine phosphoribosyltransferase	*xpt*	XX999_02513	EC:2.4.2.22	GH10
	tRNA-guanosine34 transglycosylase	*tgt*	XX999_01714	EC:2.4.2.29	GH10
	triphosphoribosyl-dephospho-CoA synthase	*citG*	XX999_01169	EC:2.4.2.52	–
	Glutamine amidotransferase^∗∗^	*hisH*	XX999_02268	EC:2.4.2.-	GH10
	*S*-adenosylmethionine :tRNA ribosyltransferase-isomerase	*queA*	XX999_01135	EC:2.4.99.17	–
			XX999_02510		
			XX999_02269		
**Phosphotransferases**	Glucokinase	*glk*	XX999_01642	EC:2.7.1.2	–
	Fructokinase	*scrK*	XX999_00302	EC:2.7.1.4	–
	Rhamnulokinase	*rhaB*	XX999_03099	EC:2.7.1.5	–
	Galactokinase	*galK*	XX999_03468	EC:2.7.1.6	–
	6-phosphofructokinase	pfkA	XX999_03415	EC:2.7.1.11	–
	Gluconokinase	*gntK*	XX999_03299	EC:2.7.1.12	–
	Ribokinase	*rbsK*	XX999_01922	EC:2.7.1.15	–
	Xylulokinase	*xylB*	XX999_01285	EC:2.7.1.17	–
	1-phosphofructokinase	*fruK*	XX999_00576	EC:2.7.1.56	–
	Glycerate 2-kinase	*glxK*	XX999_02236	EC:2.7.1.165	–
	Phosphoglycerate kinase	*pgk*	XX999_03490	EC:2.7.2.3	–
	Ribose-phosphate diphosphokinase	prsA	XX999_03492	EC:2.7.6.1	–
	Glucose-1-phosphate adenylyltransferase	glgC	XX999_02075	EC:2.7.7.27	–
			XX999_03125		
			XX999_03346		
			XX999_00881		
			XX999_00563		
			XX999_02133		
			XX999_00115		
			XX999_00116		
**Glycosylases (glycosyl hydrolases)**	Oligo-1,6-glucosidase	*malL*	XX999_00306	EC:3.2.1.10	GH13, GH31
	Alpha-glucosidase^∗∗^	*malZ*	XX999_00309	EC:3.2.1.20	GH4, GH13, GH31, GH63, GH97, GH122
	Alpha-galactosidase^∗∗^	*galA*	XX999_03453	EC:3.2.1.22	GH4, GH27, GH31, GH36, GH57, GH97, GH110
	Beta-galactosidase^∗∗^	*lacZ*	XX999_03369	EC:3.2.1.23	
	Alpha-mannosidase	E3.2.1.24	XX999_03302	EC:3.2.1.24	GH1, GH2, GH3, GH35, GH39, GH42, GH50, GH59, NC
	Beta-fructofuranosidase^∗∗^	*sacA*	XX999_03300	EC:3.2.1.26	
	Xylan 1,4-beta-xylosidase^∗∗^	*xynB*	XX999_03301	EC:3.2.1.37	GH31, GH38, GH92
	Alpha-L-rhamnosidase	*ramA*	XX999_03309	EC:3.2.1.40	GH32, GH68, GH100
	Beta-*N*-acetylhexosaminidase^∗∗^	*nagZ*	XX999_03287	EC:3.2.1.52	GH1, GH3, GH5, GH30, GH39, GH43, GH51, GH52, GH54, GH116, GH120
	Cyclomaltodextrinase^∗∗^	*ma*	XX999_03438	EC:3.2.1.54	
	Non-reducing end alpha-L-arabinofuranosidase^∗∗^	*abfA*	XX999_03461	EC:3.2.1.55	GH78, GH106, CE15
	6-phospho-beta-glucosidase	*bglA*	XX999_00304	EC:3.2.1.86	GH3, GH5, GH18, GH20, GH84, GH116, NC
	Alpha, alpha-phosphotrehalase	*treC*	XX999_03314	EC:3.2.1.93	GH13, GH57
	Mannosylglycerate hydrolase				
	Alpha-D-xyloside xylohydrolase	*mngB*	XX999_02624	EC:3.2.1.170	
		*xylS*	XX999_03313	EC:3.2.1.177	GH2, GH3, GH10, GH43, GH51, GH54, GH62
			XX999_03312		GH1, GH4
			XX999_02682		GH13
			XX999_03314		GH38, GH63
			XX999_00538		
			XX999_02708		
			XX999_02709		
			XX999_02906		
			XX999_03006		
			XX999_03053		
			XX999_03350		
			XX999_03357		
			XX999_03358		
			XX999_03459		
			XX999_00377		
			XX999_03347		
			XX999_03495		
					
					GH31
**Isomerases**	Ribulose-phosphate 3-epimerase	*rpe*	XX999_01689	EC:5.1.3.1	–
	UDP-glucose 4-epimerase	*gale*	XX999_00804	EC:5.1.3.2	GT1
	Aldose 1-epimerase	*galM*	XX999_01230	EC:5.1.3.3	–
	L-ribulose-5-phosphate 4-epimerase	*araD*	XX999_02084	EC:5.1.3.4	–
	*N*-acylglucosamine-6-phosphate 2-epimerase	*nanE*	XX999_03032	EC:5.1.3.9	–
	UDP-*N*-acetylglucosamine 2-epimerase (non-hydrolyzing)	*wecB*	XX999_03298	EC:5.1.3.14	GT4
	L-rhamnose mutarotase	*rhaM*	XX999_00914	EC:5.1.3.32	–
	2-epi-5-epi-valiolone epimerase	*cetB*	XX999_01783	EC:5.1.3.33	–
	D-allulose-6-phosphate 3-epimerase	*alsE*	XX999_03304	EC:5.1.3.-	–
	Triose-phosphate isomerase	*tpiA*	XX999_03394	EC:5.3.1.1	–
	L-arabinose isomerase	*araA*	XX999_03407	EC:5.3.1.4	–
	Xylose isomerase	*xylA*	XX999_01209	EC:5.3.1.5	–
	Ribose-5-phosphate isomerase	*rpiA*	XX999_03414	EC:5.3.1.6	–
	Mannose-6-phosphate isomerase	*manA*	XX999_00348	EC:5.3.1.8	–
	Glucose-6-phosphate isomerase	*pgi*	XX999_03373	EC:5.3.1.9	–
	L-rhamnose isomerase	*rhaA*	XX999_00882	EC:5.3.1.14	–
	1-(5-phosphoribosyl)-5-[(5-phosphoribosylamino)methylideneamino]imidazole-4-carboxamide isomerase	*hisA*	XX999_03393	EC:5.3.1.16	–
		*trpF*	XX999_03493	EC:5.3.1.24	–
		*hxlB*	XX999_00477	EC:5.3.1.27	–
	Phosphoribosylanthranilate isomerase	*pgm*	XX999_00762	EC:5.4.2.2	–
	6-phospho-3-hexuloisomerase	*pgmB*	XX999_02356	EC:5.4.2.6	–
	Phosphotransferases (phosphomutases)	*glmM*	XX999_02452	EC:5.4.2.10	–
	Beta-phosphoglucomutase	*gpmA*	XX999_03413	EC:5.4.2.11	–
	Phosphoglucosamine mutase	*gpmB*	XX999_02509	EC:5.4.2.12	–
	Phosphoglycerate mutase (2,3-diphosphoglycerate-dependent)		XX999_01716		
	Phosphoglycerate mutase (2,3-diphosphoglycerate-independent)		XX999_03454		
			XX999_00856		
			XX999_00121		
			XX999_00179		
			XX999_00910		
			XX999_00758		
			XX999_03037		
			XX999_00318		
			XX999_00974		
			XX999_00975		
			XX999_01026		
			XX999_01833		
			XX999_02136		
			XX999_02714		
			XX999_02790		


***^∗^**Last update 17/02/2017. ^∗∗^Carbohydrate-binding module (CBM) proteins. NC, non-classified; GH, Glycoside Hydrolase; GT, Glycosyl Transferase; CE, Carbohydrate Esterase.*

Furthermore, the presence of sugar ABC transporters, carbohydrate esterases, glycosyl transferases, polysaccharide lyases, permeases, and PEP-PTS (PEP; PTS) components required for the uptake and metabolism of plant and host-derived carbohydrates were predicted in the *L. pentosus* MP-10 genome, as similarly reported for the probiotic *Bifidobacterium* (Kim et al., 2009). This arsenal of genes coding for carbohydrate-modifying enzymes predicted in *L. pentosus* MP-10 genome could represent a key indicator of this bacterium’s adaptation to the GIT environment, as these genes are involved in the metabolism and transport of carbohydrates non-digestible by human enzymes. Glycosyl (hexosyl-, pentosyl-, and phospho-) transferases are involved in the biosynthesis of disaccharides, oligosaccharides and polysaccharides by transferring sugar moieties from an activated donor to a specific substrate (Lairson et al., 2008); the resulting glycoconjugates (as part of the glycome) play an important role in the establishment of environment- and host-specific interactions (Kay et al., 2010). Glycoside hydrolases are able to hydrolyze the glycosidic bond between two or more carbohydrates, and also between carbohydrate and non-carbohydrate moieties. The most common predicted genes found in *L. pentosus* MP-10 were coding for oligo-1,6-glucosidase, beta-galactosidase, alpha-L-rhamnosidase, and 6-phospho-beta-glucosidase among others (with several GH families), playing a key role not only in carbohydrate hydrolysis but also their action as retaining enzymes involved in the synthesis of oligosaccharides that may be selectively used as prebiotics by *L. pentosus* MP-10 and other gastrointestinal probiotic bacteria (**Table 1**).

Regarding isomerases, we observed several carbohydrate isomerases involved in the glycolytic pathway; however, the presence of different copies of phosphoglycerate mutase may indicate that gene-products may accomplish other functions as a moonlighting protein (Candela et al., 2007).

### Complex Carbohydrate Metabolism

*Lactobacillus pentosus* MP-10 has the capacity to metabolize complex carbohydrates (e.g., starch, cellulose, galactan, xylan, pullulan, pectins, and gums). For example, glycogen metabolism plays an important role in survival and fitness of LAB in their ecological niche by contributing to cellular processes such as carbohydrate metabolism, energy production, stress response, and cell–cell communication (Eydallin et al., 2007, 2010). The glycogen metabolism operon (*glg*) predicted in *L. pentosus* MP-10 is encoded by a 9608-base chromosomal region and consists of *glgBCDAP-apu g*enes (XX999_00114 to XX999_00119), which are co-transcribed as polycistronic mRNA (**Table 2**). The organization of the core genes (*glgBCDAP*) is identical to many bacteria, with the exception of two additional glycogen synthase genes exclusive to *L. pentosus* MP-10 (XX999_01233 and XX999_02081) which are homologous with *Bacillus subtilis* 168 and *Mycobacterium tuberculosis* CDC 1551, respectively (**Table 2**). Furthermore, genes *amyB* and *pgcA* coding for alpha-amylase 2 and phosphoglucomutase, respectively, are distantly located from the *glg* operon (**Table 2** and **Figure 2B**). According to Goh and Klaenhammer (2014), the glycogen gene cluster organization might differ depending on the bacterial species and origin; in this study, the glycogen gene cluster is composed of *glgBCDAP-apu-amyB-pgcA* genes and the other two glycogen synthase genes (XX999_01233 and XX999_02081). Glycogen metabolism is predicted as an additional trait in *L. pentosus* MP-10, as it will contribute to probiotic activities and the retention of this bacterium in highly competitive and dynamic niches, such as the gastrointestinal environment, similarly as the probiotic *L. acidophilus* (Goh and Klaenhammer, 2013). The presence of more than one glycogen synthase gene in *L. pentosus* MP-10 indicates the capacity of these bacteria to store carbohydrates in the form of glycogen.

**Table 2:** Genes necessary for the glycogen metabolism in *Lactobacillus pentosus* MP-10 isolated from naturally fermented Aloreña table olives.

Gene ID	Gene	Gene length (bp)	Protein (Uniref_protein)	GO terms
XX999_00114	*glgB*	1623	1,4-alpha-glucan branching enzyme GlgB (UniRef100:P30538)	1,4-alpha-glucan branching enzyme activity (MF); hydrolase activity, hydrolyzing O-glycosyl compounds (MF); glycogen biosynthetic process (BP); cation binding (MF)
XX999_00115	*glgC*	1140	Glucose-1-phosphate adenylyltransferase (UniRef100:P39122)	ATP binding (MF); glycogen biosynthetic process (BP); glucose-1-phosphate adenylyltransferase activity (MF)
XX999_00116	*glgD*	1173	Glycogen biosynthesis protein GlgD (UniRef100:P39124)	Glycogen biosynthetic process (BP); nucleotidyltransferase activity (MF)
XX999_00117	*glgA*	1440	Glycogen synthase (UniRef100:P39125)	Glycogen biosynthetic process (BP); starch synthase activity XX999_00297
XX999_00118	*glgP*	2403	Glycogen phosphorylase (UniRef100:P39123)	Glycogen metabolic process (BP); glycogen phosphorylase activity (MF); pyridoxal phosphate binding (MF)
XX999_00119	*apu*	1818	Amylopullulanase precursor (UniRef100:P16950)	Starch binding (MF); alpha-amylase activity (MF); carbohydrate metabolic process (BP); metal ion binding (MF); pullulanase activity (MF)
XX999_00297	*amyB*	1323	Alpha-amylase 2 (UniRef100:P14898)	Alpha-amylase activity (MF); cytoplasm (CC); carbohydrate metabolic process (BP); metal ion binding (MF)
XX999_00856	*pgcA*	1728	Phosphoglucomutase (UniRef100:P18159)	Magnesium ion binding (MF); phosphoglucomutase activity (MF); cytosol (CC); glycogen biosynthetic process (BP); glucose metabolic process (BP); enterobacterial common antigen biosynthetic process (BP); galactose catabolic process (BP)
XX999_01233	*XX999_01233*	1032	Glycogen synthase (UniRef100:P9WMY8)	Glycogen (starch) synthase activity (MF); glycogen biosynthetic process (BP)
XX999_02081	*XX999_02081*	1041	Glycogen synthase (UniRef100:P9WMY8)	Glycogen (starch) synthase activity (MF); glycogen biosynthetic process (BP)


*BP, Biological process; CC, Cellular component; MF, Molecular function.*

**FIGURE 3:**
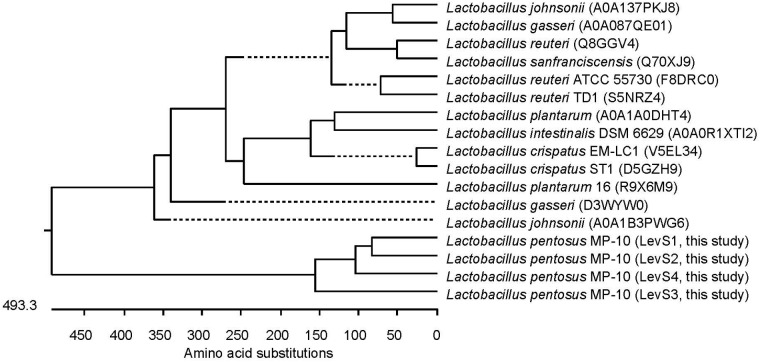
**Phylogenetic relationships of *L. pentosus* MP-10 and other *Lactobacillus* sp. inferred from the alignment of levansucrase proteins.** The sequences were aligned and the most parsimonious phylogenetic trees were constructed using the CLUSTAL W of Lasergene program, version 14 (MegAlign 14, Inc., Madison, WI, USA). The scale below indicates the number of amino acid substitutions. Accession numbers are indicated in parentheses.

*Lactobacillus pentosus* MP-10 possesses genes predicted as levansucrase (*levS_1, levS_2, levS_3*, and *levS_4*) with identities ranging from 44.07 to 62.4% with *levS* gene from *L. sanfranciscensis* (**Table 3**; Rhee et al., 2002; Tieking et al., 2005), which are responsible for levan polymers [fructan polymers composed of β(2,6)-linked fructose units] and the fructo-oligosaccharide (FOS) 1-kestose production with prebiotic effects. This bacterium is capable to produce levan [with β-2,6 glycosidic bonds, produced by levansucrases (E.C. 2.4.1.10)] but not inulin-fructan types as no inulosucrase genes were detected in *L. pentosus* MP-10 genome. This is the first report of levansucrase in *L. pentosus*; this enzyme has only been reported in other LAB (*L. sanfranciscensis L. reuteri*, *L. johnsonii*, *L. gasseri*, *L. crispatus*, *L. plantarum*, *L delbrueckii*, and *L. vaginalis* among others). Alignments of the amino acid sequence of LevS proteins of *L. pentosus* MP-10 (LevS1, LvS2, LevS3, and LevS4) with levansucrase proteins of other lactic acid bacteria revealed less similarity and formed a separate cluster in the phylogenetic tree (**Figure 3**).

**Table 3:** Genes necessary for complex carbohydrate metabolism in *Lactobacillus pentosus* MP-10 isolated from naturally fermented Aloreña table olives.

Carbohydrate	Gene ID	Gene	Gene length (bp)	Protein (Uniref_protein)	Identity (%)	E-value	GO terms
Levan	XX999_02538	*levS_1*	2448	Levansucrase (UniRef100:Q70XJ9)	44.07	2e-07	Extracellular region (CC); cell wall (CC); carbohydrate metabolic process (BP); carbohydrate utilization (BP); metal ion binding (MF); levansucrase activity (MF)
	XX999_02724	*levS_2*	3078	Levansucrase (RefSeq:Q70XJ9)	46.67	3e-24	
	XX999_02966	*levS_3*	2688	Levansucrase (UniRef100:Q70XJ9)	50.4	2e-06	Extracellular region (CC); cell wall (CC); membrane (CC)
	XX999_02983	*levS_4*	6552	Levansucrase (UniRef100:Q70XJ9)	62.4	1e-09	Extracellular region (CC); cell wall (CC); carbohydrate metabolic process (BP); carbohydrate utilization (BP); metal ion binding (MF); levansucrase activity (MF)
Chitin	XX999_00964	*XX999_00964*	759	Hypotheticalprotein	26.87	8e-23	Polysaccharide catabolic process (BP); cytoplasm (CC); chitin catabolic process (BP); chitin disaccharide deacetylase activity (MF); metal ion binding (MF); diacetylchitobiose catabolic process (BP)
	XX999_03477	*exo I*	1851	Beta-hexosaminidase (UniRef100:P96155)	25.73	8e-12	Polysaccharide catabolic process (BP); beta-N-acetylhexosaminidase activity (MF); chitin catabolic process (BP); periplasmic space (CC)
Raffinose	XX999_03302	*rafA*	2217	Alpha-galactosidase (UniRef100:P16551)	33.16	4e-96	Carbohydrate metabolic process (BP); raffinose alpha-galactosidase activity (MF)
Cellulose	XX999_00850	*XX999_00850*	1446	Cellulose synthase regulator protein (CLUSTERS:PRK11114)	–	–	–
	XX999_00851	*XX999_00851*	702	Cellulose synthase regulator protein (CLUSTERS:PRK11114)	–	–	–
	XX999_01507	*bcsA*	1986	Cellulose synthase catalytic subunit [UDP-forming] (UniRef100:P37653)	27.89	3e-65	Plasma membrane (CC); UDP-glucose metabolic process (BP); integral component of membrane (CC); cellulose synthase (UDP-forming) activity (MF); cyclic-di-GMP binding (MF); bacterial cellulose biosynthetic process (BP)
	XX999_02472	*yedQ*	1194	Putative diguanylate cyclase YedQ (UniRef100:P76330)	28.91	7e-20	Negative regulation of bacterial-type flagellum-dependent cell motility (BP); GTP
	XX999_03259	*XX999_03259*	984	Hypothetical protein (UniRef100:P10477)	24.64	3e-06	lipid metabolic process (BP); cellulase activity (MF); hydrolase activity, acting on ester bonds (MF); cellulose catabolic process (BP)
Xylan	XX999_00089	*XX999_00089*	588	Polysaccharide deacetylase (UniRef100:P54865)	30.77	7e-05	Hydrolaseactivity, actingoncarbon-nitrogen (butnotpeptide) bonds (MF); polysaccharidebinding (MF); endo-1,4-beta-xylanase activity (MF); xylancatabolicprocess (BP)
	XX999_01054	*axeA1_1*	798	Acetylxylan esterase precursor (UniRef100:D5EV35)	26.82	2e-11	Xylancatabolicprocess (BP); acetylxylan esterase activity (MF)
	XX999_02525	*xynY*	918	Endo-1,4-beta-xylanase Y precursor (UniRef100:P51584)	29.51	3e-29	Endo-1,4-beta-xylanase activity (MF); cellulosome (CC); xylancatabolicprocess (BP)
	XX999_03401	*axeA1_2*	837	Acetylxylan esterase precursor (UniRef100:D5EV35)	27.63	4e-12	Xylancatabolicprocess (BP); acetylxylan esterase activity (MF)
	XX999_03577	*axeA1_3*	714	Acetylxylan esterase precursor (UniRef100:D5EV35)	27.59	3e-12	Xylancatabolicprocess (BP); acetylxylan esterase activity (MF)


*BP, Biological process; CC, Cellular component; MF, Molecular function.*

Regarding other enzymes involved in complex carbohydrate degradation, we found genes coding for a protein similar to chitooligosaccharide deacetylase of *E. coli* K12 and beta-hexosaminidase involved in chitin degradation pathway as part of glycan degradation. Further, several genes coding for enzymes involved in the degradation of plant structural polysaccharides such as cellulose, ß-glucan, and xylan were predicted in *L. pentosus* MP-10 genome (**Table 3**). In this context, a gene coding for a protein similar to cellulase/esterase CelE from *Clostridium thermocellum* ATCC 27405, which is a multifunctional enzyme involved in the degradation of plant cell wall polysaccharides, was identified in *L. pentosus* MP-10 genome necessary for cellulose and xylan digestion by both human and animals (**Table 3**). Moreover, endo-1,4-beta-xylanase, acetylxylan esterase (three genes) and polysaccharide deacetylase were predicted in *L. pentosus* MP-10 genome sequence being involved in xylan catabolic pathway. Alpha-galactosidase coding gene was also detected in *L. pentosus* MP-10 genome sequence and is involved in raffinose degradation (**Table 3**), which was previously shown *in vitro* by Pérez Montoro et al. (2016). Furthermore, *L. pentosus* MP-10 also had genes coding for cellulose synthase (two genes exclusive to *L. pentosus* MP-10 and two other genes) involved in cellulose synthesis (**Table 3**), which could accumulate cellulose on the cell wall surface as an extracellular matrix for cell adhesion and biofilm formation to protect the bacteria. Cellulose production has been reported in lactic acid bacteria (Adetunji and Adegoke, 2007); however, no reports were found of cellulase production, although some *Lactobacillus* sp. genomes exhibit cellulase genes such as *L. delbrueckii* subsp. *bulgaricus* CNCM I-1519 (UniProtKB-G6F519) and *L. plantarum* (UniProtKB – A0A1C9HK74). For probiotic bacteria, such as *E. coli* Nissle 1917, cellulose production is required for adhesion of bacteria to the gastrointestinal epithelial cell line HT-29, to the mouse epithelium *in vivo*, and for enhanced cytokine production (Monteiro et al., 2009). Thus, the role of cellulose production in *L. pentosus* MP-10 must be investigated in depth.

Overall, the repertoire of enzymes coding genes identified in *L. pentosus* MP-10 genome highlight the attractiveness of this bacterium as potential probiotic for human and animal.

### Molecular Mechanisms Involved in the Interaction with the Host

Probiotic lactobacilli can mimic the same mechanisms used by the pathogens in the colonization process, thus they can express cell surface proteins such as key probiotic ligands that interact with host receptors resulting in several probiotic effects—thus inducing signaling pathways in the host (Voltan et al., 2008). The identification and characterization of these proteins, often strain-specific, involved in the molecular communication or interaction with the host are necessary to evaluate *a priori* the probiotic potential of *Lactobacillus* sp. candidates. Here, the possible interaction between *L. pentosus* MP-10 and the intestinal host cells, the target of most interactions with probiotics (Lebeer et al., 2010), may be bioinformatically predicted from the genome sequence. For example, several extracellular proteins (reviewed by Sánchez et al., 2008) were predicted in *L. pentosus* MP-10 to be involved in mucus adhesion: MucBP domain protein (codified by two genes determined in this study), lipoprotein signal peptidase (*lspA* gene) and moonlighting proteins such as glutamine-binding periplasmic protein (*glnH* genes) and elongation factor Tu (*tuf* gene) (**Table 4**). The high genetic heterogeneity of MucBP proteins among *Lactobacillus* species (and strains) was reported by Mackenzie et al. (2010) for MUB and MUB-like proteins in *L. reuteri*. MucBP domain proteins are bacterial peptidoglycan-bound proteins, which are ligands or effector molecules contributing to specific properties such as adherence to the host, auto-aggregation and/or co-aggregation with pathogenic bacteria (Pérez Montoro et al., 2016)—as reported by Mackenzie et al. (2010) for MUB in *L. reuteri*. However, this should be further investigated for *L. pentosus* MP-10 under different conditions. Adhesion to mucus has been attributed to other molecules such as the *Lactobacillus* surface protein A (LspA), reported as mucus binding protein in *L. salivarius* UCC118 (van Pijkeren et al., 2006), which was also found in *L. pentosus* MP-10 (**Table 4**). Mucus binding proteins in *L. pentosus* MP-10 may have a dual role: (1) being involved in the adhesion of this bacterium to the host cells and thus reinforcing the protection of the mucosal barrier and the competitive exclusion of pathogens, and (2) these proteins could also be implicated in the induction of mucin secretion by the host as reported for other lactobacilli (Mack et al., 2003). These finding are corroborated by the fact that *L. pentosus* MP-10 was able to adhere to Caco-2 and HeLa 229 cell lines and also co-aggregate with different pathogens (*Escherichia coli*, *Staphylococcus aureus*, *Listeria innocua*, and *Salmonella* Enteritidis) (Pérez Montoro et al., 2016) by means of cell-wall surface molecules. However, further studies are required to demonstrate the target cell-wall surface molecules involved in such adhesion to intestinal cells.

**Table 4:** Genes coding for extracellular proteins with roles in adhesion or interaction with the host as predicted from genome annotation of *Lactobacillus pentosus* MP-10 isolated from naturally fermented Aloreña table olives.

Gene ID	Gene	Gene length (bp)	Protein (Uniref_protein/Pfam)^∗^	Identity (%)	E-value	Organism	GO terms
XX999_01369	*XX999_01369*	11817	MucBP domain protein (Pfam:PF06458.6)	–	–	–	Mucin-Binding Protein
XX999_01708	*XX999_01708*	6885
XX999_00892	*glnH_1*	1437	Glutamine-binding periplasmic protein	40.98	5e-43	*Escherichia coli* O157:H7	Transporter activity (MF); amino acid transport (BP); periplasmic space (CC)
XX999_02287	*glnH_3*	840	precursor (UniRef100:P0AEQ5)	31	1e-29
XX999_01827	*lspA*	450	Lipoprotein signal peptidase (UniRef100:C4ZPV3)	55.5	1e-10	*Escherichia coli* K12	Aspartic-type endopeptidase activity (MF); plasma membrane (CC); integral component of membrane (CC)
XX999_02097	*tuf*	1188	Elongation factor Tu (UniRef100:P0DA82)	77.08	0.0	*Streptococcus pyogenes* ATCC BAA-595	Translation elongation factor activity (MF); GTPase activity (MF); GTP binding (MF); cytoplasm (CC)
XX999_01594	*pgaC_1*	1314	Poly-beta-1,6-N-acetyl-D-glucosamine	33.89	3e-66	*Escherichia coli* K12	Plasma membrane (CC); metabolic process (BP); acetylglucosaminyltransferase activity (MF); integral component of membrane (CC); cell adhesion involved in biofilm formation (BP)
XX999_02115	*pgaC_2*	1356	synthase (UniRef100:P75905)	25.97	1e-19
X999_01138	*psaA_1*	942	Manganese ABC transporter substrate-binding	51.96	6e-113	*Streptococcus pneumoniae* ATCC BAA-334	Plasma membrane (CC); cell adhesion (BP); metal ion transport (BP); metal ion binding (MF)
XX999_02913	*psaA_2*	894	binding lipoprotein precursor	27.21	7e-23
XX999_03164	*psaA_3*	909	(UniRef100:P0A4G2)	25.09	4e-13		
XX999_00883	*eno2*	1329	Enolase 2 (UniRef100:Q042F4)	78.65	0.0	*Lactobacillus gasseri* ATCC 33323	Phosphopyruvate hydratase complex (CC); magnesium ion binding (MF); phosphopyruvate hydratase activity (MF); extracellular region (CC); glycolytic process (BP); cell surface (CC)
XX999_00880	*gap*	1023	Glyceraldehyde-3-phosphate dehydrogenase (UniRef100:Q59309)	57.86	2e-137	*Clostridium pasteurianum*	Glyceraldehyde-3-phosphate dehydrogenase (NAD++) (phosphorylating) activity (MF); cytoplasm (CC); glucose metabolic process (BP); glycolytic process (BP); NADP binding (MF); NAD binding (MF)
XX999_02862	*XX999_02862*	1884	Collagen binding domain protein	–	–	–	–
XX999_00818	*groS*	285	10 kDa chaperonin (UniRef100:Q07200)	61.96	6e-37	*Geobacillus stearothermophilus*	ATP binding (MF); cytoplasm (CC); protein folding (BP)
XX999_00819	*groL*	1626	60 kDa chaperonin (UniRef100:Q04IQ3)	75.79	0.0	*Staphylococcus aureus* Mu50	ATP binding (MF); cytoplasm (CC); protein refolding (BP)
XX999_01649	*pgiglnA*	1347	Glutamine synthetase (UniRef100:P60890)	67.86	0.0	*Streptococcus pneumoniae* D39	Glutamate-ammonia ligase activity (MF); ATP binding (MF); cytoplasm (CC); glutamine biosynthetic process (BP); nitrogen fixation (BP)
XX999_02452	*pgi*	1353	Glucose-6-phosphate isomerase (UniRef100:P81181)	64.96	0.0	*Lactococcus lactis* subsp. *lactis* IL1403	Glucose-6-phosphate isomerase activity (MF); cytoplasm (CC); gluconeogenesis (BP); glycolytic process (BP)


*BP, Biological process; CC, Cellular component; MF, Molecular function.*

Other proteins predicted to be involved in adhesion to epithelial cells or extracellular matrix include: poly-beta-1,6-*N*-acetyl-D-glucosamine synthase, collagen binding protein, manganese ABC transporter substrate-binding lipoprotein precursor and moonlighting proteins such as elongation factor Tu, glyceraldehyde-3-phosphate dehydrogenase, 10 and 60 kDa chaperonins, enolase, 2 glutamine synthetase, and glucose-6-phosphate isomerase (**Table 4**). The poly-beta-1,6-*N*-acetyl-D-glucosamine synthase encoded by *L. pentosus* MP-10 was similar to *E. coli* K12 (33.89% identity), and it has been reported to be a surface polysaccharide involved in biofilm formation by this strain (Matthysse et al., 2008). However, the role of this protein in lactobacilli has not been determined. Furthermore, we predicted the presence of collagen-binding protein specific to *L. pentosus* MP-10, which could be involved in their adhesion to epithelial cells/extracellular matrix proteins similarly as shown other lactobacilli such as *L. reuteri* NCIB 11951 (Roos et al., 1996) and *L. fermentum* RC-14 (Heinemann et al., 2000). Thus, this could be of vital importance for effective colonization and also competitive displacement of gut pathogens (Yadav et al., 2013).

On the other hand, the manganese ABC transporter substrate-binding lipoprotein precursor predicted in *L. pentosus* MP-10, similar to *Streptococcus pneumoniae* ATCC BAA-334 (51.96% identity), has been described as an important factor in pathogenesis and infection, since it acts as an adhesin involved on adherence to extracellular matrix (Dintilhac et al., 1997). Furthermore, the manganese ABC transporter substrate-binding lipoprotein precursor has also been detected in different *Lactobacillus* sp. such as *L. plantarum*, *L.* rhamnosus, and *L. delbrueckii* among others being involved in cell adhesion (UniprotKB).

The moonlighting proteins, or multifunctional proteins such as elongation factor Tu and chaperonin GroEL, have been involved in the adhesion to epithelial cells and/or extracellular matrix proteins and also in host immunomodulation in *L. johnsonii* NCC 533 (Granato et al., 2004; Bergonzelli et al., 2006; Sánchez et al., 2008), while α-enolase has been involved in adhesion to epithelial cells and/or extracellular matrix proteins and also plasma components in *L. crispatus* ST1 (Antikainen et al., 2007). Glyceraldehyde-3-phosphate dehydrogenase and phosphoglycerate mutase have been involved in the adhesion to plasma components in *L. crispatus* ST2 (Antikainen et al., 2007; Candela et al., 2007). Furthermore, Kainulainen et al. (2012) showed that glutamine synthetase and glucose-6-phosphate isomerase have also been involved in adhesion to epithelial cells. However, the role of these moonlighting proteins in *L. pentosus* MP-10 has not yet been determined, requiring for this purpose further mutation or proteomic studies.

## Conclusion

*Lactobacillus pentosus* MP-10 has harbored in its genome several genes putatively involved in their adaptation to the human GIT—particularly those involved in carbohydrate metabolism related to prebiotic utilization, and also the proteins involved in the interaction with host tissues. Enzymes involved in carbohydrate modification and complex-carbohydrate metabolism are highly represented in *L. pentosus* MP-10 genome, which may enhance their survival, competitiveness, and persistence in a competitive GIT niche. Furthermore, we found genes encoding mucus-binding proteins—involved in the adhesion to mucus, epithelial cells or extracellular matrix, to plasma components—and also moonlighting proteins, or multifunctional proteins, predicted to be involved in their adhesion to epithelial cells and/or extracellular matrix proteins and also involved in host immunomodulation. In conclusion, *in silico* analysis of the *L. pentosus* MP-10 genome sequence highlights the attractiveness of this bacterium as a potential probiotic for human and animal hosts, and offers opportunities for further investigation of novel routes for screening and studying these bacteria.

## Materials and Methods

### Genomic DNA Sequence of *L. pentosus* MP-10

The complete genome sequence of *L. pentosus* MP-10, obtained by using PacBio RS II technology (Abriouel et al., 2016) and deposited at the EMBL Nucleotide Sequence Database under accession numbers FLYG01000001 to FLYG01000006, was annotated as described by Abriouel et al. (in press). Briefly, the assembled genome sequences were annotated using the Prokka annotation pipeline, version 1.11 (Seemann, 2014), which predicted tRNA, rRNA, and mRNA genes and signal peptides in the sequences using Aragorn, RNAmmer, Prodigal, and SignalP, respectively (Laslett and Canback, 2004; Lagesen et al., 2007; Hyatt et al., 2010).

### *In Silico* Analysis of Carbohydrate Metabolism in *L. pentosus* MP-10

The annotated genome sequence was used to detect the putative genes involved in carbohydrate metabolism, their products, and the associated GO terms. Furthermore, the carbohydrate metabolic pathways were reconstructed by using BlastKOALA (last update March 4, 2016) as part of the KEGG (Kyoto Encyclopedia of Genes and Genome) tool in the pathway database^2^ for annotating genomes; here, we used the annotated genes predicted in *L. pentosus* MP-10 genome as the input query.

### *In Silico* Analysis of Proteins Involved in Interaction with Host

The annotated genome sequence was screened for mucus-binding proteins, proteins involved in adhesion to epithelial/extracellular matrix proteins, plasma components, and host immunomodulation as described in the literature (Roos et al., 1996; Heinemann et al., 2000; Granato et al., 2004; Bergonzelli et al., 2006; van Pijkeren et al., 2006; Antikainen et al., 2007; Candela et al., 2007; Sánchez et al., 2008; Mackenzie et al., 2010; Kainulainen et al., 2012).

## Author Contributions

HA, NB, CK, and AG drafted the manuscript. HA, NB, BPM, CC-S, APP, NCG, SC-G, and ME-M analyzed the data; All authors discussed the results, commented on the manuscript, and approved the final version.

## Conflict of Interest Statement

The authors declare that the research was conducted in the absence of any commercial or financial relationships that could be construed as a potential conflict of interest.

## Footnotes

1 http://www.bacterio.net

2 http://www.genome.jp/kegg/pathway.html
